# Dose Imprecision and Resistance: Free-Choice Medicated Feeds in Industrial Food Animal Production in the United States

**DOI:** 10.1289/ehp.1002625

**Published:** 2010-10-28

**Authors:** David C. Love, Meghan F. Davis, Anna Bassett, Andrew Gunther, Keeve E. Nachman

**Affiliations:** 1 Department of Environmental Health Sciences, Johns Hopkins Bloomberg School of Public Health, Baltimore, Maryland, USA; 2 Center for a Livable Future, Johns Hopkins University, Baltimore, Maryland, USA; 3 Animal Welfare Approved, Alexandria, Virginia, USA

**Keywords:** antibiotic resistance, antibiotics, antimicrobials, cow, feed blocks, industrial food animal production, livestock, medicated feed supplements, poultry, swine

## Abstract

**Background:**

Industrial food animal production employs many of the same antibiotics or classes of antibiotics that are used in human medicine. These drugs can be administered to food animals in the form of free-choice medicated feeds (FCMF), where animals choose how much feed to consume. Routine administration of these drugs to livestock selects for microorganisms that are resistant to medications critical to the treatment of clinical infections in humans.

**Objectives:**

In this commentary, we discuss the history of medicated feeds, the nature of FCMF use with regard to dose delivery, and U.S. policies that address antimicrobial drug use in food animals.

**Discussion:**

FCMF makes delivering a predictable, accurate, and intended dose difficult. Overdosing can lead to animal toxicity; underdosing or inconsistent dosing can result in a failure to resolve animal diseases and in the development of antimicrobial-resistant microorganisms.

**Conclusions:**

The delivery of antibiotics to food animals for reasons other than the treatment of clinically diagnosed disease, especially via free-choice feeding methods, should be reconsidered.

Animal feed is a broad term that, for animals produced for human consumption (e.g., dairy and beef cattle, hogs, layer hens, broiler chickens, turkeys), includes a diet that is specific to an animal’s species, age, and production stage, and may vary according to time of year and plant species grown (Forbes 2007). Feeds are often provided to food animals on a “free-choice” basis, which means that animals elect whether or not to eat the feed and how much of it to ingest. Feeds containing medically active ingredients, such as antibiotic and antiparasitic drugs or insecticides, that are fed on a “free-choice” basis, are designated free-choice medicated feeds (FCMF) by the U.S. Food and Drug Administration (FDA 2010h). The FDA has approved 685 drugs for medicated feed, some of which are consumed on a free-choice basis (for product list in a searchable database, see FDA 2010d).

The FDA reported that 13.1 million kg of antimicrobial drugs were sold or distributed for use in food-producing animals in 2009 (FDA 2010i). Many of the same antibiotics or classes of antibiotics used to treat clinical infections in humans are also used in industrial food animal production (IFAP) (McEwen and Fedorka-Cray 2002).

The use of FCMF in food animals has been associated with imprecise drug intake, leading to under- or overadministration of drugs (Figure 1) (Bogan and Marriner 1983; Hall 2000; Toutain et al. 2010). Overadministration of drugs may lead to animal toxicity (Guardabassi and Kruse 2008; Hall 2000) and the presence of drug residues in meat or milk, although this is rare (Burch et al. 2008). Underadministration or inconsistent administration of drugs may lead to animal treatment failure (Burch et al. 2008) or the emergence of antibiotic-resistant strains of microorganisms in food animals (Guardabassi and Kruse 2008; Khachatourians 1998; Lees et al. 2006). Antibiotic-resistant commensal and environmental bacteria can contribute to maintaining or perpetuating a reservoir of resistance genes (Chee-Sanford et al. 2009; Silbergeld et al. 2008; Wright 2007), and these bacteria can share genes for antibiotic resistance with pathogenic bacteria via horizontal gene transfer (Andremont 2000). Multiple resistance genes travel on the same mobile genetic element (e.g., plasmid), allowing one pharmaceutical to select for microorganisms that are resistant to multiple classes of antibiotics (Wright 2007). In addition to natural selection and horizontal gene transfer as mechanisms for resistance, sublethal bactericidal antibiotic use at doses below those expected to provide overt selective pressure induces mutations in bacterial genomes that may confer antibiotic resistance (Kohanski et al. 2010). Humans are exposed to antibiotic-resistant bacteria through many pathways, including direct animal contact (Price et al. 2007a), contact with environmental media, such as soil, water, and air, contaminated with animal waste (Graham et al. 2009), and consumption or handling of contaminated food products from animals raised with antibiotics (FDA 2010e; Johnson et al. 2009).

Use of medicated feed has been demonstrated to introduce residual antimicrobials and their metabolites into the waste streams of animal operations. As much as 75% of administered antibiotics (Chee-Sanford et al. 2009), and considerable fractions of some antiparasitic medications (Lumaret and Errouissi 2002; Wall and Strong 1987), are not absorbed by animals and are eliminated in waste. Some insecticides and antiparasitic drugs included in certain feed supplements are designed to be excreted by animals to control insects attracted to animal droppings (Wall and Strong 1987). These wastes contribute to drug loads in watersheds and in other environmental media that may become available for human or nontarget organism exposure (Arikan et al. 2008; Chee-Sanford et al. 2009; Lumaret and Errouissi 2002). One route for drug exposure to humans is through food crops that take up antibiotics when fertilized with animal manure containing these same antibiotics (Kumar et al. 2005). The human health consequences of exposures to these drugs and their metabolites at environmentally relevant concentrations remain largely uncharacterized.

The FDA regulates medicated feed (FDA 2010a, 2010b) and in doing so separates animal drugs into two types: therapeutic drugs that are used to “diagnose, cure, mitigate, treat, or prevent disease in animals,” and animal production drugs that are intended only for healthy livestock and used to “enhance the production of edible or nonedible products or to increase the efficiency of a particular phase of life” to increase the rate of weight gain, improve feed efficiency, or enhance milk production (Sechen 2006). Both types of drugs, by FDA regulation, can be sold without veterinary prescription as medicated feed products (FDA 2000, 2010c). As part of the FDA approval, all medicated feed products are required to have labels listing active ingredients by medication name and concentration, indications for use, and all other ingredients by weight so that contents can be read and interpreted by workers at animal production facilities. In some cases, a medicated feed is broadly labeled both for disease treatment of sick animals and for disease prevention or growth promotion in healthy animals.

The purpose of this commentary is to discuss the history of medicated feed, the nature of FCMF use, and its role in the development of antimicrobial-resistant microorganisms. We also discuss the past and current legislative efforts to address antimicrobial use in food animals.

## A History of Medicated Feed

The rationale for including antibiotics in feed was constructed in the late 1940s and early 1950s when studies began to report a correlation between the use of antibiotics (mainly chlortetracycline and oxytetracycline) in livestock, swine, and poultry with increased rates of animal weight gain (Jones and Ricke 2003). The food animal production industry implemented in-feed antibiotic use in conjunction with high-throughput single-species cultivation to enhance production of grain-fed, feedlot livestock and poultry (Pew Commission on Industrial Farm Animal Production 2008). Antibiotic and antihelminthic additives were included in mineral and other feed supplements beginning in the early 1960s and 1970s, respectively (Hanson 1963). In 1960, the Agricultural and Medical Research Council Committee of Great Britain suggested that antibiotics offered economic advantages to livestock producers by lowering production costs (Kiser 1976).

Concerns were raised as early as the 1950s when researchers noted that antibiotic-resistant bacteria emerged when animals were administered antibiotics (Finland 1956). The potential for adverse human health consequences resulting from low-level exposures to antibiotic residues in meat, milk, and eggs also was identified (Randall 1956). Apprehension regarding the use of human drugs in animals was noted in a 1969 European report, *Use of Antibiotics in Animal Husbandry and Veterinary Medicine* (Swann 1969). U.S. regulators addressed these concerns in a 1972 report by the FDA Task Force on the Use of Antibiotics in Animal Feeds (Van Houweling and Gainer 1978) and an FDA-proposed policy statement (Edwards 1972). In 1972 and 1973, strong reaction against the proposed FDA policy statement from representatives of the livestock industry, veterinary pharmaceutical manufacturers, and some animal science researchers prompted revision of the final 1974 policy to incorporate consideration of the value of antibiotics in animal feed for increased rate of gain, feed efficiency, and disease control (Gardner 1973; Kiser 1976). Industry groups have continued to influence the regulatory trajectory of policies surrounding the use of antimicrobials in food animal production.

## Antimicrobial Dose Delivery by FCMF

Difficulties in ensuring precision in antimicrobial dose delivery, a function of numerous factors, may result in the inability to deliver predictable, uniform, or intended dose levels. These factors, although not mutually exclusive, can be grouped into concerns regarding labeling, veterinary oversight, feed characteristics, the behavior of animal production facility workers, animal behavior, and drug pharmacokinetics.

### Quality control of medicated feed

Requirements for quality control in manufacturing for antibiotics intended for use in animal finished feed products are less rigorous than those for pharmaceuticals intended for parenteral use in livestock or for human use (FDA 2009, 2010f, 2010g). Quality control problems can occur when drugs are combined with other feed ingredients. In an investigation of feeds labeled as nonmedicated, 44% (71 of 161) actually contained antimicrobials, and more than one-third (87 of 247) of feeds labeled as medicated contained undeclared antimicrobials, most notably chlortetracycline, sulfonamides, penicillin, and ionophores (Lynas et al. 1998). Investigators hypothesized that the presence of these unlabeled drugs was a result of cross-contamination in feed production mills. Quality control issues in animal feed content are not limited to those containing medication—a 2008 commercial feed survey by the U.S. Department of Agriculture (USDA) found that nearly 20% (123 of 657) of animal feed products were mislabeled; their product ingredient claims were not substantiated by an independent testing laboratory (USDA 2008).

The undeclared presence of medications in feed supplements results in the unintended delivery of antimicrobials and could compromise the USDA organic certification status of food animal products. Allowance of antibiotic use in organic breeder stock (organic broiler chicks before and on the first day of hatching) as well as in herds used for cattle replacement of organic dairy cattle may undermine the spirit of USDA organic certification.

### Animal production facility worker behavior

Because veterinary oversight or prescription is not required to purchase, mix, or administer most medicated feed products (FDA 2000), workers at animal production facilities may be exclusively responsible for the decision to use medicated feed. Certain medicated feeds are labeled to treat specific morbidities, for example, anaplasmosis or *Pasteurella* pneumonia (FDA 2010d). Such diseases often require clinical diagnosis and laboratory confirmation (Hartwig and Ensley 2010). Allowing workers to purchase medicated feed without a veterinary prescription to treat diseases that require a veterinarian to diagnose may result in drug administration in a manner inconsistent with its intended use.

### Animal and herd behavior

The number of animals per feeding location or feed block, feed location within animal production facility, other housing or grazing area characteristics and herd or flock social interactions are factors shown to influence feed intake for ruminants and poultry (Appleby 1998; Bowman and Sowell 1997, 2002; Kincheloe 2004). Interanimal dominance scenarios influence the quantity of feed consumed by ruminants (Bowman and Sowell 2002), and in poultry, pecking order affects medicated feed intake, leading to differences in drug exposure among animals (Toutain et al. 2010). Acceptance of feed increases with animal age and prior experience with feed type (Bowman and Sowell 1997; Kincheloe 2004). A fraction of livestock herds refuse FCMF; these animals were shown to have low serum and plasma concentrations of drugs (Bogan and Marriner 1983). Administering medication via FCMF is not an efficient way to achieve accurate serum and plasma concentrations of drugs across a herd or flock of animals.

### Nondomesticated animals

When FCMF are freely available to livestock, they may be available to wildlife living on or near animal production operations. Studies in Europe and the United States have demonstrated that antibiotic-resistant bacteria can be found in feral hogs and other wildlife (Costa et al. 2008; Literak et al. 2010; Ramlachan et al. 2007). Wildlife living on farms are more likely to harbor antibiotic-resistant *Escherichia coli* than wildlife not living on farms (Kozak et al. 2009). Although exposure to feed may explain transfer of resistant bacteria to wildlife, other possible production-related pathways of exposure exist, including contact with contaminated manure, dust, or water. Wildlife movement from the source farm rarely is restricted and can spread antimicrobial-resistant microorganisms and diseases to other communities or farms (Ramlachan et al. 2007; Ward et al. 2006).

### Drug absorption, pharmacokinetics, and pharmacodynamics

After drug consumption, factors that influence absorption and pharmacokinetics determine target organ dose (Lees et al. 2006). Some drugs given orally, such as tetracyclines, may bind to cations (e.g., calcium) that are found in feed, lowering their bioavailability and resulting in serum concentrations below minimum inhibitory concentrations, even in animals dosed at levels for disease treatment (Nielsen and Gyrd-Hansen 1996; Underhill and Danish 1992). Proteins, carbohydrates, and fats present in feeds also may alter absorption of many drugs (Underhill and Danish 1992). Commensal bacteria present in the rumen of cows may chemically alter drugs administered orally (Then 1982). Finally, disease states alter an animal’s ability to absorb and process drugs for systemic delivery (Guardabassi and Kruse 2008). Fever, concurrent organ damage (e.g., hepatic lipidosis in dairy cows), and gastrointestinal disease may influence the final serum concentrations of a drug (Kozak et al. 2009; Underhill and Danish 1992). This is of particular concern when medications in feed supplements are licensed for disease treatment; the animals most in need of a drug may be the ones least likely or able to access doses consistent with disease treatment.

Taken in sum, numerous factors influence the ability to deliver predictable or intended doses of drugs to animals via FCMF. Given the limited oversight, the availability of FCMF without a veterinary prescription, the potential for undeclared drugs, and variability in drug concentrations within and between feeds, unintended (and therefore inappropriate) drug delivery is likely. At a minimum, these factors make predicting the actual delivered dose to any given individual animal nearly impossible and predicting herd averages for drug delivery complicated. Worse, inappropriate or imprecise drug dosing may drive selection for resistant microorganisms (Lees et al. 2006), that affect both veterinary and human medicine.

## Policy Considerations

### U.S. federal legislation

Elimination of antibiotics for growth promotion and feed efficiency in IFAP in the United States has been discussed since the 1970s. The latest antibiotics bill was reintroduced by Congresswoman Louise Slaughter (D-NY) in 2009 as the Preservation of Antibiotics for Medical Treatment Act (PAMTA; H.R.1549/S. 619, 2009). PAMTA would withdraw federal approval for use of certain drugs as feed or water additives in food animal production if they are used for growth promotion, feed efficiency, or weight gain, and for disease prevention in the absence of any clinical sign of disease in an animal. A former head of the FDA, Donald Kennedy, called for Congress to pass PAMTA (Kennedy 2010). Passage of the bill would not affect the use of insecticides (e.g., tetrachlorvinphos or methoprene) or antiparasitic drugs (e.g., thiabendazole) in medicated feeds that are fed for therapeutic uses, or antiparasitic drugs for nontherapeutic uses. Although regulating the use of antiparasitics and insecticides is relevant to the ecology of drug resistance, these drugs are more appropriate for oral dosing using herd health management methods. Some evidence supports efficacy of antiparasitics, insecticides, and certain antibiotics in particular species via feed supplement administration (Blagburn et al. 1987; McBride et al. 2008).

Although PAMTA begins to address these concerns, more effort is needed to reform the conventional (nonorganic) U.S. dairy, beef cattle, hog, and poultry industries. In Denmark, swine and poultry productivity stabilized and, in some cases, increased after bans on nontherapeutic uses of certain antibiotics during the late 1990s (Aarestrup 2009; Lawson et al. 2008). Denmark monitors the veterinary use of antimicrobial drugs for IFAP through VETSTAT, a transparent data reporting system that tracks compliance of legislation, helps veterinarians work with animal production facility workers, and provides data for research on veterinary drugs (Stege et al. 2003). In the United States, no federal requirements currently exist for reporting animal antimicrobial drug use by animal production facility staff or veterinarians, although in a historic move, the FDA began reporting annual antimicrobial drug distribution and sales summary data in 2010 as required by the 2008 reauthorization of the Animal Drug User Fee Act (FDA 2010i). If these data are made publically available in a timely fashion and usable format, then an assessment of the extent of antibiotic use in IFAP can be conducted. Although reporting itself will not mitigate antimicrobial resistance risks, understanding the extent, nature, and distribution of antimicrobial use in IFAP will strengthen the impetus for policy interventions aimed at eliminating unnecessary administration of antimicrobials.

### U.S. federal regulatory agency involvement

Human cases of fluoroquinolone-resistant, food-borne *Campylobacter* increased after the licensing of fluoroquinolones for use in poultry during the 1990s (Gupta et al. 2004; Nelson et al. 2007). As a result, the FDA withdrew approval for fluoroquinolone use in water to treat diseases in poultry in 2005 in response to concerns over agricultural drivers of resistant infections in humans (Gupta et al. 2004; Nelson et al. 2007). Even after the fluoroquinolone ban, bacterial resistance persisted in poultry products (Price et al. 2007b), highlighting the importance of early action to remove nontherapeutic use of antibiotics in livestock. In 2010, the FDA issued a draft guidance over concerns with antimicrobial use, stating that “the overall weight of evidence available to date supports the conclusion that using medically important antimicrobial drugs for production purposes is not in the interest of protecting and promoting the public health” (FDA 2010j). However, as guidance, the FDA document is not binding or enforceable.

## Conclusion

Delivering antibiotics to food animals for reasons other than treatment of clinically diagnosed disease, especially via free-choice feeding methods, poses an unnecessary public health risk. Mounting evidence suggests the use of antibiotics in food animal production contributes to a considerable fraction of antibiotic-resistant infections in humans (Silbergeld et al. 2008). The increasing number of antimicrobial-resistant infections and their costs in the United States, estimated to be $400 million to $5 billion per year in 1998 (Institute of Medicine 1998) and $16.6 to $26 billion per year in 2009 (Roberts et al. 2009), are of growing concern. Methicillin-resistant *Staphylococcus aureus* (MRSA) infections acquired outside of hospitals (i.e., community-associated MRSA) have seen a 33% annual increase from 1999 to 2006 (Klein et al. 2009). Although not all community-associated MRSA infections originate from IFAP, certain human cases have been associated with the production of swine, poultry, and dairy cattle in Europe (Broens et al. 2008), and the potential for similar exposure pathways exists in the United States. Human exposures to antibiotic- resistant *Campylobacter*, *Salmonella*, and other resistant bacteria via food animal products are also of concern (Angulo et al. 2004; Kassenborg et al. 2004; Price et al. 2005).

Instead of farming practices that employ free-choice oral herd or flock dosing with medicated feed, appropriate individual therapeutic antimicrobial administration by injection should be pursued to treat clinically diagnosed disease in food animals. Therapeutic treatment by injection will achieve more predictable plasma drug levels, enhancing opportunities for disease control (Baggot 2007; Lees et al. 2006). In cases where individual treatment is difficult, such as with large poultry operations, improving husbandry and hygiene at production facilities are preferred over the administration of oral therapeutic antimicrobial with veterinary supervision, and all are preferable to FCMF without veterinary supervision. For prevention of diseases in a herd or flock, vaccinations (Redding and Weiner 2009), reduced stress (Dantzer and Mormède 1983), and improved welfare (Broom 2006) are approaches that can help replace antimicrobials.

## Figures and Tables

**Figure 1:**
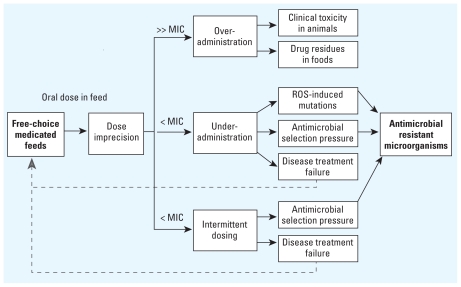
Dose imprecision for food animals that consume FCMF and a pathway to antibiotic resistance. Overadministration leads to very high plasma or target tissue levels of antibiotic. Underadministration leads to levels of antibiotic that never reach minimum inhibitory concentrations (MICs). Intermittent dosing leads to levels of antibiotic that fluctuate and periodically dip below MICs for variable periods of time. ROS, reactive oxygen species. Dotted lines indicate positive feedback, potentially driving increased use of FCMF.
